# Supplementary material for: Association between oral microbiome and seven types of cancers in East Asian population: a two-sample Mendelian randomization analysis
Source: Front Mol Biosci. 2023 Nov 21;10:1327893. doi: 10.3389/fmolb.2023.1327893 (PMC10702768; doi:10.3389/fmolb.2023.1327893)
Supplement: Supplementary file 1 [file DataSheet1.ZIP › Supplementary Materials/MR plots of saliva/saliva═╝/Prostate cancer/pheno.2859_to_prostate cancer_forest.pdf]

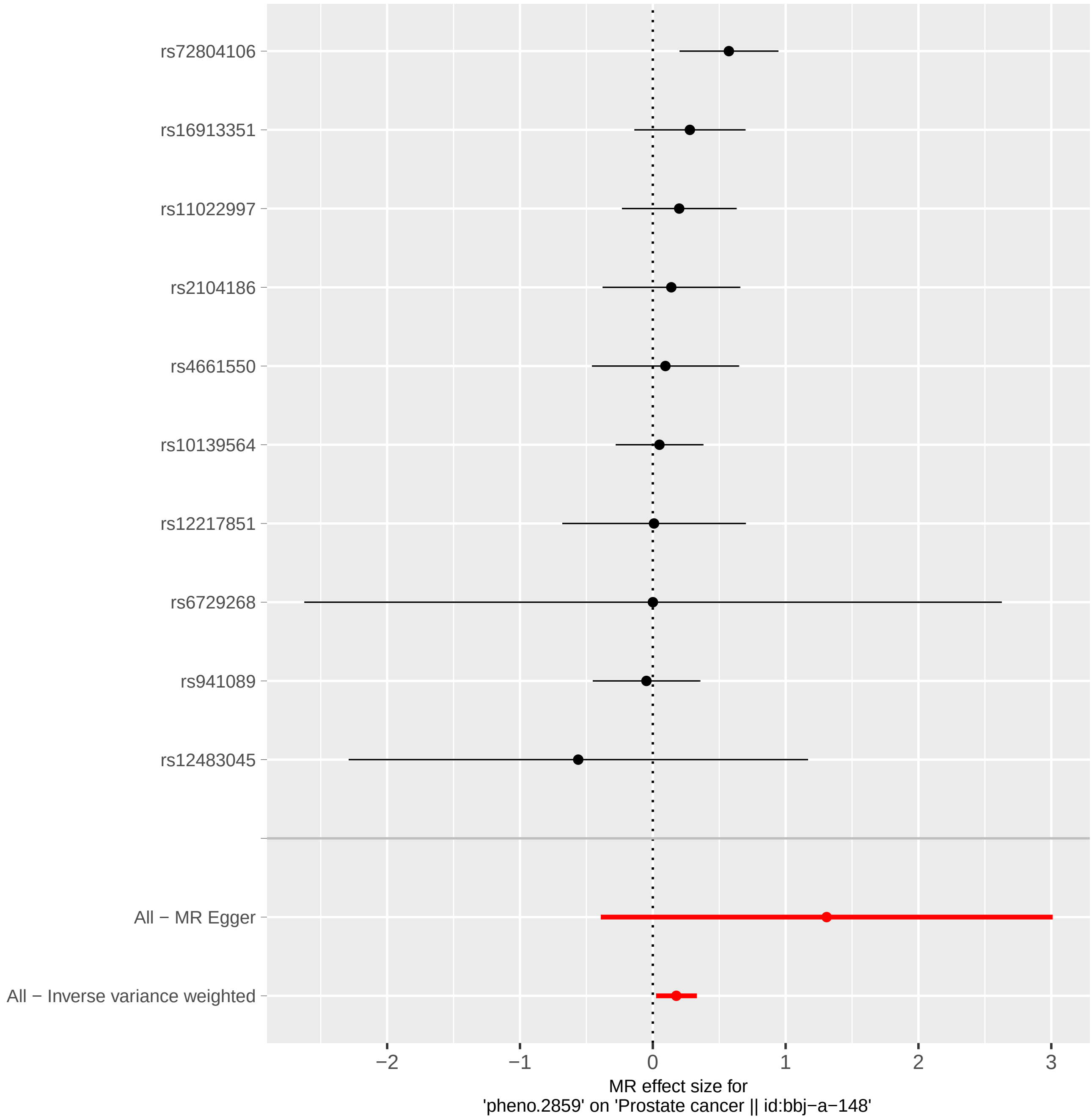
rs72804106
rs16913351
rs11022997
rs2104186
rs4661550
rs10139564
rs12217851
rs6729268
rs941089
rs12483045
All − MR Egger
All − Inverse variance weighted
−2
−1
0
1
2
3
MR effect size for
'pheno.2859' on 'Prostate cancer || id:bbj−a−148'
